# Circulating Fatty Acids and Risk of Coronary Heart Disease and Stroke: Individual Participant Data Meta‐Analysis in Up to 16 126 Participants

**DOI:** 10.1161/JAHA.119.013131

**Published:** 2020-03-02

**Authors:** Maria Carolina Borges, Amand Floriaan Schmidt, Barbara Jefferis, S. Goya Wannamethee, Debbie A. Lawlor, Mika Kivimaki, Meena Kumari, Tom R. Gaunt, Yoav Ben‐Shlomo, Therese Tillin, Usha Menon, Rui Providencia, Caroline Dale, Aleksandra Gentry‐Maharaj, Alun Hughes, Nish Chaturvedi, Juan Pablo Casas, Aroon D. Hingorani, the UCLEB Consortium**

**Affiliations:** ^1^ MRC Integrative Epidemiology Unit at the University of Bristol United Kingdom; ^2^ Population Health Sciences Bristol Medical School University of Bristol United Kingdom; ^3^ Institute of Cardiovascular Science University College London London United Kingdom; ^4^ Groningen Research Institute of Pharmacy University of Groningen the Netherlands; ^5^ Division Heart and Lungs Department of Cardiology University Medical Center Utrecht Utrecht The Netherlands; ^6^ UCL Department of Primary Care & Population Health UCL Medical School London United Kingdom; ^7^ Department of Epidemiology and Public Health University College London London United Kingdom; ^8^ Institute for Social and Economic Research University of Essex United Kingdom; ^9^ Cardiometabolic Phenotyping Group Institute of Cardiovascular Science University College London London United Kingdom; ^10^ MRC Clinical Trials Unit at UCL Institute of Clinical Trials & Methodology University College London London United Kingdom; ^11^ Farr Institute of Health Informatics University College London London United Kingdom; ^12^ Barts Heart Centre St Bartholomew's Hospital Barts Health NHS Trust London United Kingdom; ^13^ Massachusetts Veterans Epidemiology Research and Information Center (MAVERIC) VA Boston Healthcare System Boston MA USA

**Keywords:** coronary artery disease, epidemiology, fatty acids, stroke, Cardiovascular Disease, Epidemiology, Risk Factors, Diet and Nutrition, Primary Prevention

## Abstract

**Background:**

We aimed at investigating the association of circulating fatty acids with coronary heart disease (CHD) and stroke risk.

**Methods and Results:**

We conducted an individual‐participant data meta‐analysis of 5 UK‐based cohorts and 1 matched case‐control study. Fatty acids (ie, omega‐3 docosahexaenoic acid, omega‐6 linoleic acid, monounsaturated and saturated fatty acids) were measured at baseline using an automated high‐throughput serum nuclear magnetic resonance metabolomics platform. Data from 3022 incident CHD cases (13 104 controls) and 1606 incident stroke cases (13 369 controls) were included. Logistic regression was used to model the relation between fatty acids and odds of CHD and stroke, adjusting for demographic and lifestyle variables only (ie, minimally adjusted model) or with further adjustment for other fatty acids (ie, fully adjusted model). Although circulating docosahexaenoic acid, but not linoleic acid, was related to lower CHD risk in the fully adjusted model (odds ratio, 0.85; 95% CI, 0.76–0.95 per standard unit of docosahexaenoic acid), there was evidence of high between‐study heterogeneity and effect modification by study design. Stroke risk was consistently lower with increasing circulating linoleic acid (odds ratio for fully adjusted model, 0.82; 95% CI, 0.75–0.90). Circulating monounsaturated fatty acids were associated with higher CHD risk across all models and with stroke risk in the fully adjusted model (odds ratio, 1.22; 95% CI, 1.03–1.44). Saturated fatty acids were not related to increased CHD risk in the fully adjusted model (odds ratio, 0.94; 95% CI, 0.82–1.09), or stroke risk.

**Conclusions:**

We found consistent evidence that linoleic acid was associated with decreased risk of stroke and that monounsaturated fatty acids were associated with increased risk of CHD. The different pattern between CHD and stroke in terms of fatty acids risk profile suggests future studies should be cautious about using composite events. Different study designs are needed to assess which, if any, of the associations observed is causal.

**Clinical Perspective**

**What Is New?**

- There is inconsistent evidence on the relation of fatty acids with coronary heart disease (CHD) and stroke risk.
- We performed a large individual‐participant data meta‐analysis to assess the association of several circulating fatty acids with CHD and stroke risk and to investigate potential sources of inconsistency in the current literature.

**What Are the Clinical Implications?**

- We found consistent evidence that circulating linoleic acid is associated with lower risk of stroke and that monounsaturated fatty acids are related to higher risk of CHD.
- Our findings do not support that the association of fatty acids with CHD or stroke risk is nonlinear or modified by participants’ characteristics (ie, by age group, sex, European ancestry, body mass index, or severity of event).
- The different pattern between CHD and stroke in terms of fatty acids risk profile raises a cautionary note for studies in which composite events are used.

## Introduction

Early interest in the relation of dietary fatty acids with cardiovascular disease emerged following results from ecological studies conducted between the 1950s and 1970s. These studies indicated that populations with high consumption of saturated fatty acids (SFAs) had increased rates of mortality from cardiovascular diseases,^1^ while reduced cardiovascular disease mortality was observed in populations with high consumption of omega‐3 polyunsaturated fatty acids.^2^, ^3^ Numerous subsequent controlled feeding studies showed that dietary fatty acid composition could modulate the concentration of the major circulating blood lipid fractions (mainly total, low‐density lipoprotein cholesterol, and high‐density lipoprotein cholesterol and triacylglycerols).^4^ These observations played a central role in the classical diet‐heart hypothesis and have prompted recommendations on dietary fatty acid intake for primary and secondary prevention of cardiovascular diseases.^5^, ^6^, ^7^

In the past few decades, however, some long‐lasting beliefs concerning the effects of fatty acids on health have been challenged. For example, it has been argued that the effect of reduction of dietary SFAs on coronary heart disease (CHD) may depend on what dietary components they replace (carbohydrates or polyunsaturated fatty acids).^8^ In addition, systematic reviews of randomized controlled trials (RCTs) reported heterogeneous results regarding the protective effect of omega‐3 fatty acids on cardiovascular outcomes with more recent trials failing to replicate initial positive findings.^9^, ^10^, ^11^, ^12^, ^13^ The reasons for inconsistent findings remain elusive, although several methodological issues may have contributed to heterogeneity in both RCTs and observational studies. In the case of omega‐3, a recent systematic review of RCTs that lasted at least 12 months (n=112 059 participants) concluded that increasing omega‐3 intake has little or no effect on cardiovascular events or mortality and found no evidence of differential effects according to omega‐3 dose, trial duration, and primary or secondary prevention.^13^ For other fatty acids, evidence is less convincing.^14^ Furthermore, most studies have focused on CHD as the outcome; hence, the relevance of most fatty acids for cerebrovascular disease remains unclear.

We therefore performed an individual‐participant data meta‐analysis to assess the association of several circulating fatty acids with both CHD and stroke risk by (1) including data from 6 UK‐based studies with a focus on primary events and information on multiple confounders; (2) using objectively measured blood fatty acid concentration, which reflects dietary intake and biological processes (eg, absorption and metabolism); (3) investigating potential methodological and biological sources of heterogeneity (sex, ethnicity, age, obesity, type of event, and study design); and (4) exploring nonlinear dose‐response relationships.

## Methods

### Study Population

Individual data were available on 23 518 participants from 6 UK‐based studies (5 cohorts and 1 nested case‐control study) participating in the UCL‐LSHTM‐Edinburgh‐Bristol (UCLEB) consortium^15^: BWHHS (British Women's Heart and Health Study),^16^ BRHS (British Regional Heart Study),^17^ WHII (Whitehall‐II Study),^18^ the SABRE (Southall and Brent Revisited)^19^ cohort, CaPS (Caerphilly Prospective Study),^20^ and a case‐control study nested in the UKCTOCS (United Kingdom Collaborative Trial of Ovarian Cancer Screening)^21^ (Table 1). All participating studies obtained informed consent from participants and received ethical approval. Because of the sensitive nature of the data collected for this study, requests to access UCLEB data sets from qualified researchers trained in human subject confidentiality protocols may be sent to the UCLEB steering committee at a.hingorani@ucl.ac.uk.^15^

**Table 1:** Characteristics of Participating Studies

Study	Study Design	Original Population	Available Data	Outcome Data Collection	Website
BWHHS^16^	Cohort	4286 women aged 60–79 y recruited from general practices in 23 towns across the United Kingdom	Baseline: 2000 Follow‐up: 12 y^b^	Biennial medical record review (with validation checks) Death certificates obtained from the National Health Service (NHS) Central Registration	http://www.ucl.ac.uk/british-womens-heart-health-study
BRHS^17^	Cohort	7735 men aged 40–59 y recruited from general practices in 24 towns across the United Kingdom	Baseline: 1998–2000 Follow‐up: 10 y^b^	Biennial medical record review (with validation checks) Death certificates obtained from the NHS Central Registration	http://www.ucl.ac.uk/pcph/research-groups-themes/brhs-pub
WHII^18^	Cohort	10 308 civil servants recruited from the United Kingdom	Baseline: 1997–1999 Follow‐up: 12 y^b^	Self‐reported nonfatal events (at all phases) supplemented by information on coronary events identified by research clinic ECGs, and through verification of primary care and hospital records	http://www.ucl.ac.uk/whitehallII
SABRE^19^	Cohort	4857 individuals from European, Indian Asian and African Caribbean ancestry aged 40–69 y recruited from workplaces and general practices in the United Kingdom	Baseline: 1988–1990 Follow‐up: 25 y^b^	Identified from primary‐care and hospital records and from participant questionnaire responses Death certificates obtained from the NHS Central Registration	http://www.sabrestudy.org/
CaPS^20^	Cohort	2512 men aged 45–59 y from the town of Caerphilly and adjoining villages	Baseline: 1989–1993 Follow‐up: 12 y^b^	Self‐reported nonfatal events were validated from medical records and fatal events were collected from death certificates	http://www.bris.ac.uk/social-community-medicine/projects/caerphilly/about/
UKCTOCS^21^	Nested case‐control	1617 CHD and 863 stroke‐matched female cases selected for a case‐control study nested in a randomized controlled trial of ovarian cancer screening^a^	Baseline: 2001–2005 Follow‐up: 10 y^b^	Nonfatal and fatal events collected from medical records and death certificates, respectively	http://www.instituteforwomenshealth.ucl.ac.uk/womens-cancer/gcrc/ukctocs

BRHS indicates British Regional Heart Study; BWHHS, British Women's Heart and Health Study; CaPS, Caerphilly Prospective Study; CHD, coronary heart disease; SABRE, Southall and Brent Revisited cohort; UKCTOCS, United Kingdom Collaborative Trial of Ovarian Cancer Screening; WHII, Whitehall‐II Study.

a Maximum follow‐up length in years.

b Cases were matched with controls (known not to have experienced cardiovascular events before 2010) by age, recruitment center, and time of blood sampling.

### Ascertainment of Coronary Heart Disease and Stroke

CHD was defined as incident nonfatal myocardial infarction, revascularization procedure (coronary artery bypass surgery or angioplasty), or fatal CHD (*International Classification of Diseases, Tenth Revision* (*ICD‐10*) codes I20–I25, I51.6). Stroke was defined as incident nonfatal ischemic, hemorrhagic stroke (excluding transient ischemic attack) or fatal stroke (*International Classification of Diseases, Tenth Revision* (*ICD‐10*) codes I60.x, I61.x, I62, I62.9 I63.x, I64.x, I65.x, I66.x, I67, I67.2, I67.8, I67.9, I69.x, G46.x, G45.0, G45.1, G45.2, G45.3) (Table 1). Subjects with a prior history of CHD or stroke were excluded from analyses.

### Assessment of Fatty Acids

Fatty acids were assayed using an automated high‐throughput serum nuclear magnetic resonance metabolomics platform that quantifies 233 metabolic markers, including fatty acids, lipoprotein subclasses, α‐1 acid glycoprotein, amino acids, glycolysis‐related measures, and ketone bodies (Nightingale Health Ltd., Helsinki, Finland). The fatty acids available in the nuclear magnetic resonance platform used in the current study were the long‐chain omega‐3 docosahexaenoic acid 22:6 (DHA), the omega‐6 linoleic acid 18:2 (LA), total monounsaturated fatty acids (MUFAs), and total SFAs. The quantified fatty acids correspond to all forms of fatty acids present in the circulation (ie, all the fatty acids in triglycerides, phospholipids, or cholesterol esters, or as free fatty acids). Nuclear magnetic resonance data preprocessing and quantification were as described previously^22^, ^23^, ^24^, ^25^, ^26^, ^27^ and in Data S1.

### Covariates

Baseline information at the time fatty acids were measured included demographic variables (sex, age [in years], and ethnicity [European, Indian Asian, African Caribbeans, or other]); and lifestyle characteristics (measured body mass index [BMI; kg/m^2^], smoking [never or ever], and alcohol drinking [never or ever]). Data on smoking and alcohol drinking at baseline were not available for UKCTOCS nested case‐control study.

### Statistical Analysis

All fatty acids measures were converted to study‐ and sex‐specific *Z* scores (ie, standardized and centered to have a mean of 0 and a unit of 1 standard deviation). A 2‐stage individual participant data meta‐analysis was used to assess the association of each standardized fatty acid concentration with study covariates (ethnicity, age, smoking, alcohol drinking, and BMI); depending on the measurement scale (binary or continuous) study‐specific linear or logistic regression model were used.

Two‐stage individual participant data meta‐analysis was also used to estimate the association of baseline concentration of each fatty acid with the risk of CHD and stroke. In the first stage, logistic regression was used to model the exposure‐outcome association for each study (except for UKCTOCS, for which conditional logistic regression was used to account for matching by age, recruitment center, and time of blood sampling). In the second stage, study‐specific estimates were combined using DerSimonian & Laird random effects model.^28^ Three different models were fitted: unadjusted model (M0); minimally adjusted model (M1) (ie, adjusted for recruitment place and typical demographic/lifestyle confounders: age, non‐European background, smoking, alcohol drinking, and BMI); fully adjusted model (M2) (ie, adjusted for variables in M1 plus other fatty acids). Adjustment of M2 by the other fatty acids aimed at accounting for the correlation in the concentration of different fatty acids, allowing us to explore the independent effect of each fatty acid. Some studies have expressed fatty acids in terms of their proportion over total fatty acids. However, this approach complicates the interpretation of findings as changes in the proportion of each fatty acid could be related to both numerator and denominator of the ratio.^29^

Heterogeneity was assessed by I^2^ values. To explore the presence of effect modification on the association of fatty acids levels and CHD or stroke risk, we carried out subgroup analyses according to sex, European ethnicity (yes or no), age (<65 or ≥65 years old), BMI (underweight/normal [<25 kg/m^2^] or overweight/obese [≥25 kg/m^2^]), type of event (fatal or nonfatal), and study design (cohort or case control). To account for multiple testing, we used Bonferroni‐corrected *P* values (0.05/48=0.001) considering 48 independent tests (6 covariates×4 fatty acids×2 outcomes).

We also explored the presence of nonlinear effects by modeling CHD or stroke risk in relation to standardized fatty acids concentration using multilevel logistic regression models with restricted cubic splines with 5 knots at percentiles 5, 25, 50, 75, and 95 of study‐specific *Z* scores. Because of the matched case‐control design, UKCTOCS data were modeled separately using conditional logistic regression models with restricted cubic splines. Nonlinear models were compared with linear models using a likelihood ratio test. Bonferroni‐adjusted *P*<0.003 (0.05/16 tests) were adopted as evidence of nonlinearity. The same nonlinear models were fitted using unstandardized fatty acids concentration (mmol/L) as a sensitivity analysis.

For all models, we conducted complete case analysis (7392 and 8543 individuals were excluded from CHD and stroke models, respectively), followed by multiple imputation using Stata version 12 (StataCorp, College Park, TX). To investigate whether the missing completely at random assumption is likely to hold, we evaluated the association of our complete case indicator (0=any missing; 1=no missing variables) with baseline predictors of CHD and stroke, fatty acids, and CHD/stroke risk using logistic regression. Imputation of missing values was carried out to explore potential biases by missing data. Multivariate imputation using chained equations for 20 complete data sets with 10 iterations each was used. Imputation was performed separately for each study and included sex, age, European background, BMI, smoking, alcohol drinking, blood lipids, glucose, systolic and diastolic blood pressure, prevalent type 2 diabetes mellitus, and use of lipid‐lowering medication (some variables were not available for UKCTOCS; no participant was on lipid‐lowering medication in SABRE at baseline). Coefficients and standard errors for the variability between imputations were combined according to Rubin's rules.^30^ The same models as for the complete case analysis (M0 to M2) were fitted for each study using the imputed data sets, and study‐specific estimates were combined using the DerSimonian & Laird random‐effects model.^28^

## Results

### Descriptive Analysis

The proportion of participants who experienced CHD or stroke events was 17.1% and 9.7%, respectively, with maximum follow‐up duration ranging from 10 to 25 years across studies (Tables 1 and 2). At baseline, participants were 52.1% male, 89.1% of European background, and 61.1% aged 65 or younger. Overall, SFAs were the most abundant blood fatty acid class (mean, 4.5 mmol/L; SD, 1.2), followed by LA (mean, 3.2 mmol/L; SD, 0.8), MUFA (mean, 3.1 mmol/L; SD, 1.0) and DHA (mean, 0.2 mmol/L; SD, 0.1) (Table 2). The distribution of circulating fatty acids according to case‐control status for CHD and stroke can be found in Tables S1 and S2, respectively.

**Table 2:** Baseline Characteristics of Participants^a^ and Incident CHD and Stroke Events

	BWHHS	BRHS	WHII	SABRE	CaPS	UKCTOCS	Total
	%						
N (range)	3294–3753	3556–3796	4094–5306	2856–4442	1216–1337	1721–4884	17 782–23 518
Male	0.0	100.0	71.7	74.6	100.0	0.0	52.1
Nonwhite	0.4	0.6	0.0	51.9	0.0	3.4	10.9
Age >65 y	70.9	71.8	8.8	2.5	30.4	56.3	38.9
Smokers	11.7	13.1	16.2	45.3	82.0	···	26.6
Alcohol drinkers	38.7	63.2	91.0	82.5	93.0	···	73.4
Overweight/obese	71.9	69.8	57.5	61.2	···	60.2	59.7
Incident CHD event	8.4	9.0	3.8	19.2	24.5	50.1	17.1
Incident stroke event	5.8	6.2	1.6	8.8	13.8	50.1	9.7
	Mean (SD)						
DHA, mmol/L	0.3 (0.1)	0.2 (0.1)	0.2 (0.1)	0.1 (0)	0.1 (0)	0.2 (0.1)	0.2 (0.1)
LA, mmol/L	3.8 (0.8)	3 (0.7)	3.3 (0.6)	2.8 (0.6)	2.5 (0.7)	3.2 (0.7)	3.2 (0.8)
MUFAs, mmol/L	3.1 (1.1)	3 (0.9)	3 (0.9)	2.4 (0.7)	2.7 (1)	3.6 (1.1)	3.1 (1)
SFAs, mmol/L	4.9 (1.2)	4.2 (0.9)	4.6 (1)	3.8 (1.5)	4 (1)	4.8 (1.2)	4.5 (1.2)

BRHS indicates British Regional Heart Study; BWHHS, British Women's Heart and Health Study; CaPS, Caerphilly Prospective Study; CHD, coronary heart disease; DHA, docosahexaenoic acid; LA, linoleic acid; MUFAs, monounsaturated fatty acids; SABRE, Southall and Brent Revisited cohort; SFAs, saturated fatty acids; UKCTOCS, United Kingdom Collaborative Trial of Ovarian Cancer Screening; WHII, Whitehall‐II Study.

a Subjects with prevalent cardiovascular disease at baseline were excluded.

### Association of Circulating Fatty Acids With Baseline Characteristics

Mean changes in circulating fatty acids (in SD units) according to the distribution of demographic and lifestyle characteristics and cardiovascular disease biomarkers are shown in Table S3. On average, non‐Europeans have higher LA (0.15 standard units; 95% CI, 0.08–0.21) and lower MUFA (−0.42; 95% CI, −0.67 to −0.17) and SFA (−0.25; 95% CI, −0.44 to −0.06); the opposite pattern was found among ever smokers (LA, −0.11; 95% CI, −0.16 to −0.07; MUFA, 0.30; 95% CI, 0.22–0.37; SFA, 0.12; 95% CI, 0.04–0.21). Alcohol drinkers had increased circulation of all fatty acids, except LA (DHA, 0.35; 95% CI, 0.20–0.49; LA, −0.08; 95% CI, −0.12 to −0.04; MUFA, 0.18; 95% CI, 0.00–0.35; SFA, 0.11; 95% CI, 0.05–0.17). Older individuals had higher mean circulating DHA (0.11; 95% CI, 0.03–0.18), while overweight/obese individuals had higher MUFA (0.37; 95% CI, 0.29–0.45) and SFA (0.26; 95% CI, 0.15–0.38).

As shown in Table S4, the correlation between fatty acids within studies was mostly moderate except for SFA, which was highly correlated with MUFA (*r*_Pearson_=0.50–0.88; *P*<0.001) and with LA (*r*_Pearson_=0.16–0.71; *P*<0.001) in most studies.

### Association of Circulating Fatty Acids With CHD and Stroke Risk

**Figure 1:**
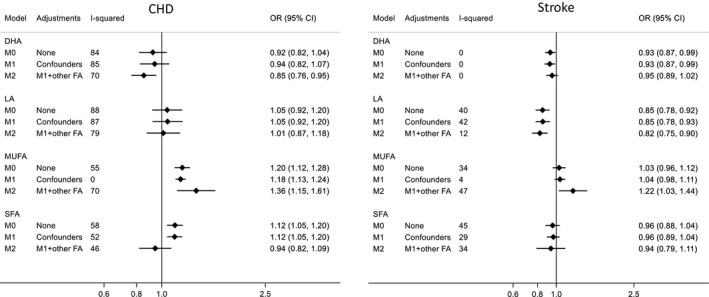
Odds ratio for coronary heart disease (CHD) and stroke according to blood fatty acids concentration. Results were pooled using random effect meta‐analysis and are expressed as odds ratio (and 95% confidence interval) per standard deviation unit increase in blood fatty acids concentration (CHD analysis, N=3022 cases and 13 104 controls; stroke analysis, N=1606 cases and 13 369 controls). Each standard unit corresponds to ≈0.06 mmol/L for DHA, 0.7 for LA, 1.0 for MUFAs, and 1.1 for SFAs. Model 0 (M0): unadjusted model; Model 1 (M1): adjusted for recruitment place, demographic and lifestyle variables (age, sex, non‐European ancestry, smoking, alcohol drinking, and body mass index); Model 2 (M2): adjusted for variables in M1 plus other fatty acids. CHD indicates coronary heart disease; DHA, docosahexaenoic acid; FA, fatty acid; LA, linoleic acid; MUFA, monounsaturated fatty acids; OR, odds ratio; SFA, saturated fatty acids.

Complete information for fatty acids–CHD and fatty acids–stroke analyses was available for 16 126 (3022 CHD cases) and 14 975 (1606 stroke cases) participants, respectively. There was some evidence of circulating DHA being associated with lower CHD risk in the fully adjusted model (odds ratio, 0.85; 95% CI, 0.76–0.95 per standard unit increase in DHA), and with stroke (OR for unadjusted/minimally adjusted models, 0.93; 95% CI, 0.87–0.99; OR for fully adjusted model, 0.95; 95% CI, 0.89–1.02). However, between‐study heterogeneity was high in CHD models for DHA (I^2^, 70%–85%) (Figure 1). Higher circulating omega‐6 LA was consistently associated with lower stroke risk in all models (eg, OR for fully adjusted model: 0.82; 95% CI, 0.75–0.90), but not associated with CHD risk. Between‐study heterogeneity was high for the association of LA with CHD risk (I^2^, 79%–88%), but moderate for its association with stroke risk (I^2^, 12%–42%) (Figure 1).

Higher blood MUFA was related to increased CHD risk in all models (eg, OR for fully adjusted model, 1.36; 95% CI, 1.15–1.61) and with increased stroke risk in the fully adjusted model only (OR, 1.22; 95% CI, 1.03–1.44). SFA levels were related to increased CHD risk in the unadjusted and minimally adjusted models only (OR, 1.12; 95% CI, 1.05–1.20) and were not related to stroke risk. Between‐study heterogeneity was variable for MUFA in both CHD (I^2^, 0%–70%) and stroke models (I^2^, 4%–47%) and moderate for SFA in both CHD (I^2^, 46%–58%) and stroke models (I^2^, 29%–45%) (Figure 1).

Further details on metrics of statistical heterogeneity in the meta‐analyses are available in Tables S5 and S6. Pooled results using fixed‐effect meta‐analysis (with inverse variance weights) are also provided for comparison (Figure S1).

### Subgroup Analysis and Nonlinear Effects

**Figure 2:**
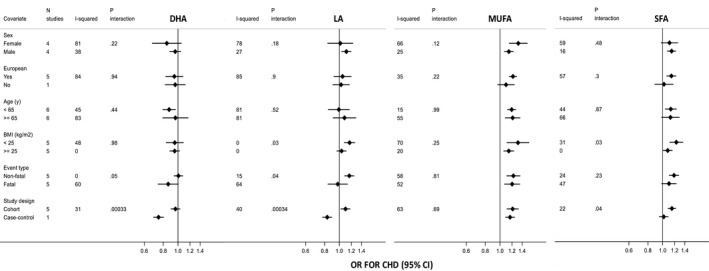
Odds ratio for coronary heart disease in relation to blood fatty acids concentration according to study covariates. Results are expressed as odds ratio (and 95% CI) per SD unit increase in blood fatty acids concentration. Model was adjusted for recruitment place, demographic and lifestyle variables (age, sex, non‐European ancestry, smoking, alcohol drinking, and body mass index). UKCTOCS was excluded from the analysis stratified by body mass index, as this was not a matching variable. Only the SABRE South Asian participants contributed to the non‐European stratum. *P* value for interaction threshold after Bonferroni correction=0.05/48=0.001. BMI indicates body mass index; CHD, coronary heart disease; DHA, docosahexaenoic acid; LA, linoleic acid; MUFA, monounsaturated fatty acids; OR, odds ratio; SFA, saturated fatty acids.

**Figure 3:**
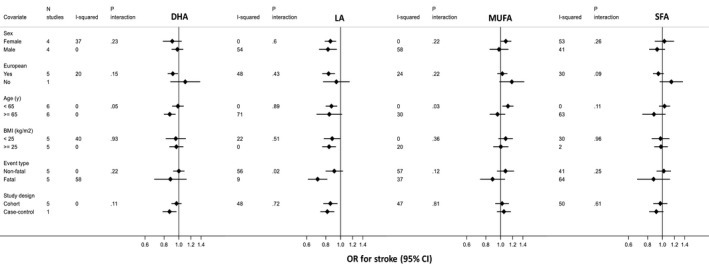
Odds ratio for stroke in relation to blood fatty acids concentration according to study covariates. Results are expressed as odds ratio (and 95% CI) per SD unit increase in blood fatty acids concentration. Model was adjusted for recruitment place, demographic and lifestyle variables (age, sex, non‐European ancestry, smoking, alcohol drinking, and body mass index). UKCTOCS was excluded from the analysis stratified by body mass index, as this was not a matching variable in the case‐control design. Only the SABRE study had enough participants to contribute to analyses in the non‐Europeans stratum. *P* value for interaction threshold after Bonferroni correction=0.05/48=0.001. BMI indicates body mass index; DHA, docosahexaenoic acid; LA, linoleic acid; MUFA, monounsaturated fatty acids; OR, odds ratio; SFA, saturated fatty acids.

In the subgroup analysis for the association between polyunsaturated fatty acids (ie, DHA and LA) and CHD risk, heterogeneity was largely explained by study design, with the case‐control study estimating a stronger protective effect compared with the null or slightly positive effect estimated by the cohorts for both DHA (*P*_interaction_=0.0003) and LA (*P*_interaction_=0.0003) (Figure 2). There was no strong evidence of effect modification by any other variable for CHD risk (Figure 2) and by any variable for stroke risk (Figure 3). In addition, there was no consistent evidence of nonlinear associations between fatty acid levels and CHD or stroke risk (Figures S2 and S3).

### Missingness

Our complete case analysis indicator was associated with CHD risk in SABRE and UKCTOCS and with stroke risk in BWHHS and UKCTOCS. The complete case indicator was also associated with several circulating fatty acids in several studies (eg, BWHHS, BRHS, and UKCTOCS) and with some of the study covariates (Table S7). The associations of fatty acid levels with CHD and stroke risk in complete case analysis (Figure 1) were broadly similar to those in the imputed data (Table S8).

## Discussion

Our findings indicate that circulating LA (omega‐6 fatty acid) and DHA (omega‐3 fatty acid) are not robustly associated with lower CHD risk. Although circulating DHA is related to lower CHD risk after adjusting for other fatty acids, there is evidence of high between‐study heterogeneity and effect modification by study design. Stroke risk was consistently lower with increasing circulating LA and slightly lower with increasing circulating DHA. Circulating MUFAs were associated with higher CHD risk across all models and with stroke risk only after accounting for other fatty acids, while SFAs were not related to increased CHD risk after accounting for other fatty acids or to stroke risk.

Omega‐3 and omega‐6 fatty acids are essential nutrients, as they cannot be produced endogenously. For this reason, blood concentration of these fatty acids reflects both dietary intake and metabolism and are increasingly being used as biomarkers of their consumption^31^ to avoid some biases from self‐reported dietary intake. A previous meta‐analysis including 25 721 participants estimated that circulating DHA (relative risk, 0.79; 95% CI, 0.67–0.93), but not LA (relative risk, 0.99; 95% CI, 0.77–1.28) was associated with lower CHD risk^12^ when comparing the top to the bottom tertile of fatty acid concentration. Fewer studies have assessed the association of blood fatty acids with stroke risk and have reported no association with circulating long chain omega‐3,^11^, ^32^, ^33^ as well as an inverse^32^, ^33^, ^34^ or null^35^ relation to LA. The 2 largest studies reported a relative risk for stroke of 1.04 (95% CI, 0.90–1.20) comparing the top tertile of long chain omega 3 fatty acids with the bottom (n=4096)^11^ and a relative risk for stroke of 0.72 (95% CI, 0.59–0.89) per 1 SD increase in LA (n=7450).^33^ Recent systematic reviews of RCTs concluded that there is probably little or no effect of increasing long‐chain omega‐3 (eg, DHA) on CHD or stroke risk^13^ and that the effect of increasing omega‐6 on CHD or stroke events is unclear because of the low quality of the evidence available.^14^

Previous studies hypothesized that a potential threshold effect of omega‐3 intake on CHD risk could explain inconsistencies between observational studies^12^ and RCTs^9^, ^10^, ^11^, ^12^ in a way that moderate consumption could be associated with benefits compared with low consumption in observational studies, while adding omega‐3 supplement doses to an already moderate background could produce smaller or no effects in RCTs.^36^ In a recent systematic review of RCTs, there was no suggestion of any dose‐response effect of long‐chain omega‐3 fatty acids on multiple cardiovascular events.^13^ In addition, we and others^36^ found no evidence of nonlinear dose‐response between circulating long‐chain omega‐3 and CHD risk. Conversely to our analyses, diet intake and circulating omega‐3 are associated in a nonlinear fashion, with steepest dose‐response in blood omega‐3 concentration with consumption of up to 400 mg/day, which might explain nonlinear trends between omega‐3 consumption and CHD risk.^36^

Even‐numbered SFAs (eg, 16:0, palmitic acid; and 18:0, stearic acid) and MUFAs (eg, 16:1 n‐7, palmitoleic acid; and 18:1 cis‐9, oleic acid) are among the most abundant in the blood and, therefore, are likely to be overrepresented in our measurement.^12^, ^37^, ^38^, ^39^ These fatty acids can be synthetized endogenously, by the process known as de novo lipogenesis. Therefore, circulating even‐numbered SFAs and MUFAs tend not to reflect dietary fatty acids intake, but nonlipid dietary precursors of de novo lipogenesis, including carbohydrate and alcohol intake. We observed that MUFAs were positively associated with CHD risk in contrast to results from a previous meta‐analysis.^12^ It is possible that these associations could also be reflecting underlying hepatic de novo lipogenesis activation, which is related to increased blood triglycerides, ectopic fat deposition, and insulin resistance.^40^, ^41^ The association of circulating SFAs with CHD risk was attenuated when adjusting for other fatty acids (model 2). However, this result should be interpreted with caution because of high correlation and interconversion between SFAs and MUFAs (eg, even‐numbered MUFAs originate from desaturation of SFAs). Smaller previous studies have reported a positive association between circulating MUFAs and SFAs and stroke risk,^32^, ^33^, ^34^ which were mostly not confirmed by our findings, although there was some evidence for a positive association between MUFAs and stroke risk after accounting for other fatty acids.

Fatty acids are dynamic molecules capable of influencing a wide range of cell signaling pathways and potentially modulating lipid metabolism,^4^, ^42^, ^43^ glucose homeostasis,^44^, ^45^ blood pressure,^46^, ^47^, ^48^ inflammatory response,^49^, ^50^, ^51^ and endothelial function.^48^, ^52^ The circulating fatty acids pool reflects the dynamic contribution of multiple metabolic pathways regulated by key hormonal signals, including adipose tissue lipolysis (the main source of free circulating fatty acids during fasting), hydrolysis of blood triglycerides in triglyceride‐rich lipoproteins, reesterification to triglycerides within adipocytes, and peripheral fatty acids utilization.^53^ In addition, fatty acids are transformed into other fatty acids by processes of desaturation and elongation. For these reasons, isolating the effect of circulating fatty acids from other pathways is challenging in studies of observational nature. In addition, lipoprotein traits not only share metabolic regulation pathways with fatty acids^53^ but might also be downstream effects of fatty acids.^4^, ^42^, ^44^, ^45^, ^46^, ^47^, ^49^

Our findings show differences between CHD and stroke in terms of their association with blood fatty acid levels. This has particularly important implications in the context of RCTs, in which the use of composite events (eg, combining CHD and stroke) is a frequent practice to increase study power but might in fact attenuate effect estimates toward the null when the effect of the treatment is specific to either CHD or stroke.

The strengths of our study include the large sample size, long follow‐up, comprehensive case ascertainment, and adjustment for many potential confounders, as well as assessment of nonlinear effects and effect modification by biological and methodological features. In addition, access to individual participant data allowed us to compare similar models across all participating studies, unlike most meta‐analyses based on summary data. Some limitations should be highlighted such as the measure of blood fatty acids at a single time point, which might have introduced nondifferential measurement error and consequently underestimated our effect estimates; the low resolution of the nuclear magnetic resonance platform with regard to identifying multiple fatty acids within each class despite evidence of heterogeneous association of individual fatty acids within omega‐3, omega‐6, and SFA classes with cardiometabolic disease^12^, ^54^; and the possibility of residual confounding attributable to the complex interplay of exogenous (diet) and endogenous (metabolism and genetics) factors, which we were unable to tease out. In addition, in the analysis of effect modification by the study covariates, some studies could only contribute with data to one of the strata of a covariable (eg, for the sex‐stratified analysis, women‐only studies such as BWHHS and UKCTOCS contributed data only for the female stratum), which might have introduced bias, as the studies differ in other characteristics.

We found consistent evidence that circulating LA is associated with lower risk of stroke and that MUFA is related to higher risk of CHD. The different pattern between CHD and stroke in terms of fatty acids risk profile raises a cautionary note for studies in which composite events are used. Different study designs (eg, clinical trials and Mendelian randomization studies) will be needed to assess which, if any, of the associations observed is causal.

## Sources of Funding

Drs Borges, Lawlor, and Gaunt work in the MRC Integrative Epidemiology Unit at the University of Bristol that receives funding from the UK Medical Research Council (MRC) (MC_UU_12013/5 and MC_UU_12013/8). Dr Borges is supported by MRC Skills Development Fellowship (MR/P014054/1). Dr Lawlor is a UK National Institute for Health Research (NIHR) Senior Investigator (NF‐SI‐0611‐10196). Dr Gentry‐Maharaj is funded by NIHR. Dr Menon is supported by the NIHR, Biomedical Research Centre at University College London Hospital. Dr Dale is supported by a University College London Springboard Population Science Fellowship (105604/Z/14/Z). The UCLEB consortium, which is supported by British Heart Foundation Programme Grants RG/10/12/28456 and SP/13/6/30554, consists of 12 studies: NPHS II (Northwick Park Heart Study II), BRHS, WHII, (ELSA) English Longitudinal Study of Ageing), MRC NSHD (Medical Research Council National Survey of Health and Development), 1958BC (1958 Birth cohort), CaPS, BWHHS, EAS (Edinburgh Artery Study), EHDPS (Edinburgh Heart Disease Prevention Study), ET2DS (Edinburgh Type 2 Diabetes Study), and AAAT (Asymptomatic Atherosclerosis Aspirin Trial). BWHHS is supported by the British Heart Foundation (PG/13/66/30442). Data on mortality and cancer events were routinely provided from NHS Digital to the BWHHS under data sharing agreement MR104a‐ Regional Heart Study (Female Cohort). British Women's Heart and Health Study data are available to bona fide researchers for research purposes. Please refer to the BWHHS data‐sharing policy at http://www.ucl.ac.uk/british-womens-heart-health-study. BRHS is supported by British Heart Foundation grants (RG/08/013/25942, RG/13/16/30528). The British Heart Foundation had no role in the design and conduct of the study; collection, management, analysis, and interpretation of the data; preparation, review, or approval of the manuscript; and decision to submit the manuscript for publication. The authors acknowledge the British Regional Heart Study team for data collection. The WHII study is supported by grants from the Medical Research Council (K013351), British Heart Foundation (RG/07/008/23674), Stroke Association, the US National Heart, Lung, and Blood Institute (5RO1 HL036310), the US National Institute on Aging (5RO1AG13196), the US Agency for Healthcare Research and Quality (HS06516), and the John D. and Catherine T. MacArthur Foundation Research Networks on Successful Midlife Development and Socio‐economic Status and Health. SABRE was supported at baseline by the UK Medical Research Council, The British Heart Foundation, and Diabetes UK. SABRE was supported at 20‐ and 25‐year follow‐up by the Wellcome Trust (WT082464) and British Heart Foundation (SP/07/001/23603 and CS/13/1/30327). Diabetes UK funded the metabolomics analyses (13/0004774). UKCTOCS was funded by the Medical Research Council (G9901012 and G0801228), Cancer Research UK (C1479/A2884), and the Department of Health, with additional support from The Eve Appeal. Phenotypic data for this case‐control data set was supported by the National Institute for Health Research, Biomedical Research Centre at University College London Hospital, who also support the biobank. CaPS was funded by the Medical Research Council and undertaken by the former MRC Epidemiology Unit (South Wales). The CaPS DNA bank was established with funding from a MRC project grant. The CaPS data archive is maintained by the University of Bristol. MRC Integrative Epidemiology Unit, Bristol is supported by MRC grants (MR_UU_12013/1, MR_UU_12013/5, and MR_UU_12013/8).

## Disclosures

Dr Lawlor has received support from Medtronic LTD and Roche Diagnostics for biomarker research that is not related to the study presented in this paper. Dr Gaunt receives support from GlaxoSmithKline, Biogen, and Sanofi for research unrelated to this study. Dr Menon has stocks in Abcodia Pvt Ltd, which has an interest in cancer biomarkers and screening. Dr Casas received grant funding from GSK to conduct methodological work on multiomics and electronic health records for drug discovery. The remaining authors have no disclosures to report.

## Supporting information


**Appendix S1.** List of additional UCLEB members.
**Data S1.** Supplemental methods.
**Table S1.** Distribution of Fatty Acids (Mean and Standard Error) According to CHD Status and Study
**Table S2.** Distribution of Fatty Acids (Mean and Standard Error) According to Stroke Status and Study
**Table S3.** Association of Blood Fatty Acids Concentration With Demographic and Lifestyle Factors
**Table S4.** Correlation Across Circulating Fatty Acids
**Table S5.** Pooled Estimates and Heterogeneity Metrics for Meta‐Analysis of the Association of Circulating Fatty Acids With Risk of CHD
**Table S6.** Pooled Estimates and Heterogeneity Metrics for Meta‐Analysis of the Association of Circulating Fatty Acids With Risk of Stroke
**Table S7.** *P* Value for the Association Between the Complete Case Indicator for CHD or Stroke Analyses and Study Variables
**Table S8.** Odds Ratio for CHD and Stroke According to Blood Fatty Acids Concentration After Multiple Imputation
**Figure S1.** Odds ratio for coronary heart disease (CHD) and stroke according to blood fatty acids concentration (fixed‐effects meta‐analysis).
**Figure S2.** Dose‐response curve for the association between blood fatty acids and coronary heart disease (CHD) risk.
**Figure S3.** Dose‐response curve for the association between blood fatty acids and stroke risk.
