# Supplementary material for: Circulating Fatty Acids and Risk of Coronary Heart Disease and Stroke: Individual Participant Data Meta‐Analysis in Up to 16 126 Participants
Source: J Am Heart Assoc. 2020 Mar 2;9(5):e013131. doi: 10.1161/JAHA.119.013131 (PMC7335585; doi:10.1161/JAHA.119.013131)
Supplement: Supplementary file 1 — Appendix S1. List of additional UCLEB members. Data S1. Supplemental methods. Table S1. Distribution of Fatty Acids (Mean and Standard Error) According to CHD Status and Study Table S2. Distribution of Fatty Acids (Mean and Standard Error) According to Stroke Status and Study Table S3. Association of Blood Fatty Acids Concentration With Demographic and Lifestyle Factors Table S4. Correlation Across Circulating Fatty Acids Table S5. Pooled Estimates and Heterogeneity Metrics for Meta‐Analysis of the Association of Circulating Fatty Acids With Risk of CHD Table S6. Pooled Estimates and Heterogeneity Metrics for Meta‐Analysis of the Association of Circulating Fatty Acids With Risk of Stroke Table S7. P Value for the Association Between the Complete Case Indicator for CHD or Stroke Analyses and Study Variables Table S8. Odds Ratio for CHD and Stroke According to Blood Fatty Acids Concentration After Multiple Imputation Figure S1. Odds ratio for coronary heart disease (CHD) and stroke according to blood fatty acids concentration (fixed‐effects meta‐analysis). Figure S2. Dose‐response curve for the association between blood fatty acids and coronary heart disease (CHD) risk. Figure S3. Dose‐response curve for the association between blood fatty acids and stroke risk. [file JAH3-9-e013131-s001.pdf]

SUPPLEMENTAL MATERIAL

Appendix S1. List of additional UCLEB members

First Name	Last Name	Affiliation
Pimphen	Charoen	Farr Institute of Health Informatics, University College London, London, United Kingdom
Jackie	Cooper	Centre for Cardiovascular Genetics, Dept. of Medicine, University College London, UK
Fotios	Drenos	Centre for Cardiovascular Genetics, Dept. of Medicine, University College London, UK
Frank	Dudbridge	Department of Health Sciences, Centre for Medicine, University of Leicester, Leicester, UK
Jorgen	Engmann	Institute of Cardiovascular Science and Farr Institute, University College London, London, United Kingdom
Ghazaleh	Fatemifar	Farr Institute of Health Informatics, University College London, London, United Kingdom
Chris	Finan	Genetic Epidemiology Group, Institute of Cardiovascular Science, University College London, UK
Victoria	Garfield	Department of Epidemiology & Public Health, UCL Institute of Epidemiology & Health Care, University College London, UK
Alun	Hughes	Cardiometabolic Phenotyping Group, Institute of Cardiovascular Science, University College London, UK
Steve	Humphries	Centre for Cardiovascular Genetics, Dept. of Medicine, University College London, UK
Elina	Hypponen	MRC Centre of Epidemiology for Child Health, Department of Population Health Sciences, UCL Institute of Child Health, University College London, UK
Diana	Kuh	MRC Unit for Lifelong Health and Ageing, London, UK
Claudia	Langenberg	Department of Epidemiology & Public Health, UCL Institute of Epidemiology & Health Care, University College London, UK
Stela	McLachlan	Centre for Population Health Sciences, The Usher Institute of Population Health Sciences and Informatics, University of Edinburgh, UK
Vincent	Plagnol	University College London Genetics Institute, Department of Genetics, Environment and Evolution, London, UK
Christine	Power	MRC Centre of Epidemiology for Child Health, Department of Population Health Sciences, UCL Institute of Child Health, London, UK
Jackie	Price	Centre for Population Health Sciences, The Usher Institute of Population Health Sciences and Informatics, University of Edinburgh, UK
Tina	Shah	Genetic Epidemiology Group, Institute of Cardiovascular Science, University College London, UK
Reecha	Sofat	Centre for Clinical Pharmacology, University College London, London, UK
Philippa	Talmud	Centre for Cardiovascular Genetics, Dept. of Medicine, University College London, UK
Ann	Walker	Centre for Cardiovascular Genetics, Dept. of Medicine, University College London, UK
Peter	Whincup	Population Health Research Institute, St George's, University of London, UK
John	Whittaker	Genetics Division, Research and Development, GlaxoSmithKline, Harlow, UK
Andrew	Wong	MRC Unit for Lifelong Health and Ageing, London, UK

Data S1. SUPPLEMENTARY METHODS

Nuclear magnetic resonance (NMR) spectroscopy platform

The quantified metabolic measures were obtained per sample of EDTA-plasma, using a 1D proton (^1H) NMR spectroscopy-based platform described previously [1-4]. Briefly, the serum samples were stored in a freezer at -80°C. The frozen samples were first slowly thawed in a refrigerator (+4°C) overnight prior to metabolomics profiling. 260 µL plasma and 260 µL sodium phosphate buffer (75 mM Na2HPO4, 0.08% sodium 3-(trimethylsilyl)propionate-2,2,3,3-d4, 0.04% sodium azide in 80%/20% H20/D2O, pH 7.4) were mixed and transferred to NMR tubes using an 8-channel, Varispan Janus liquid handling robot (PerkinElmer). NMR spectra were acquired using a Bruker Avance III HD 500MHz spectrometer with a room temperature 5mm, inverse triple resonance TXI probe and a Bruker Avance III HD 600MHz spectrometer equipped with a nitrogen-cooled triple resonance probe (CryoProbe Prodigy TCI). Both spectrometers were equipped with SampleJet auto-samplers with cooled (6°C) sample storage. Spectra were acquired using standardized parameters using three NMR experiments or 'molecular windows' to characterize lipoproteins, low molecular weight metabolites and lipids. Lipid spectra were acquired after a standardised lipid extraction procedure performed on each sample using a VIAFLO 96 channel electronic pipette (Integra Biosciences). The quantified fatty acids used in this study correspond to all forms of fatty acids present in the circulation (i.e. all the fatty acids in triglycerides, phospholipids, or cholesterol esters, or as free fatty acids). Data pre-processing and quantification were as previously described [1-4]. The NMR spectra were analysed for absolute quantification using regression models [5]. There is a high analytical consistency, in epidemiological settings, between metabolic measures quantified by the NMR metabolomics platform and the concentrations obtained from routine clinical chemistry [6], and other analytical methods, such as gas chromatography [6, 7] and enzymatic method [6], with correlations >0.9. In addition, the consistency of biomarker associations with disease incidence for metabolic traits quantified by NMR and two widely used mass spectroscopy platforms has been demonstrated [6, 7].

SUPPLEMENTARY TABLES AND FIGURES

Table S1. Distribution of fatty acids (mean and standard error) according to coronary heart disease (CHD) status and study

	**BWHHS**	**BWHHS**	**BRHS**	**BRHS**	**WHII**	**WHII**	**SABRE**	**SABRE**	**CaPS**	**CaPS**	**UKCTOCS**	**UKCTOCS**
	Mean (SD)											
	Cases	*Controls*	Cases	*Controls*	Cases	*Controls*	Cases	*Controls*	Cases	*Controls*	Cases	*Controls*
DHA (mmol/L)	0.3 (0.09)	0.3 (0.08)	0.15 (0.05)	0.16 (0.06)	0.19 (0.06)	0.19 (0.07)	0.14 (0.04)	0.14 (0.04)	0.12 (0.04)	0.11 (0.04)	0.18 (0.06)	0.2 (0.07)
LA (mmol/L)	3.77 (0.92)	3.77 (0.8)	3.02 (0.66)	2.99 (0.64)	3.45 (0.65)	3.33 (0.64)	2.8 (0.62)	2.77 (0.6)	2.6 (0.68)	2.46 (0.67)	3.09 (0.72)	3.22 (0.69)
MUFA (mmol/L)	3.44 (1.25)	3.06 (1.08)	3.1 (0.87)	3.01 (0.85)	3.26 (0.86)	2.97 (0.86)	2.47 (0.75)	2.4 (0.71)	2.85 (0.98)	2.66 (0.97)	3.72 (1.15)	3.56 (1.07)
SFA (mmol/L)	5.08 (1.37)	4.88 (1.17)	4.22 (0.93)	4.16 (0.87)	4.92 (0.93)	4.64 (0.96)	3.92 (1.66)	3.77 (1.56)	4.21 (1.01)	3.99 (1.04)	4.85 (1.19)	4.84 (1.13)

Results based on complete case analysis (N = 3,022 CHD cases and 13,104 controls). DHA: docosahexaenoic acid; LA: linoleic acid; MUFA: monounsaturated fatty acids; SFA: saturated fatty acids. BWHHS: British Women's Heart and Health Study; BRHS: British Regional Heart Study; WHII: Whitehall-II Study; SABRE: Southall And Brent REvisited cohort; UKCTOCS: United Kingdom Collaborative Trial of Ovarian Cancer Screening; CaPS: Caerphilly Prospective Study; CHD: coronary heart disease.

Table S2. Distribution of fatty acids (mean and standard error) according to stroke status and study

	BWHHS	BWHHS	BRHS	BRHS	WHII	WHII	SABRE	SABRE	CaPS	CaPS	UKCTOCS	UKCTOCS
						Mean (SD)						
	Cases	*Controls*	Cases	*Controls*	Cases	*Controls*	Cases	*Controls*	Cases	*Controls*	Cases	*Controls*
DHA (mmol/L)	0.3 (0.09)	0.3 (0.08)	0.15 (0.06)	0.16 (0.06)	0.19 (0.08)	0.19 (0.07)	0.14 (0.04)	0.14 (0.04)	0.12 (0.05)	0.11 (0.04)	0.19 (0.06)	0.19 (0.06)
LA (mmol/L)	3.74 (0.84)	3.77 (0.81)	2.87 (0.66)	3 (0.64)	3.08 (0.66)	3.34 (0.64)	2.69 (0.64)	2.79 (0.6)	2.47 (0.63)	2.5 (0.68)	3.06 (0.7)	3.19 (0.69)
MUFA (mmol/L)	3.21 (1.27)	3.08 (1.09)	2.94 (0.83)	3.02 (0.85)	2.81 (0.79)	2.98 (0.86)	2.48 (0.76)	2.41 (0.71)	2.77 (1.02)	2.7 (0.97)	3.53 (1.12)	3.48 (1.03)
SFA (mmol/L)	4.98 (1.37)	4.89 (1.18)	4.04 (0.92)	4.18 (0.87)	4.44 (0.9)	4.66 (0.96)	3.88 (2.01)	3.79 (1.51)	4.06 (0.99)	4.04 (1.05)	4.69 (1.12)	4.78 (1.07)

Results based on complete case analysis (N = 1,606 stroke cases and 13,369 controls). DHA: docosahexaenoic acid; LA: linoleic acid; MUFA: monounsaturated fatty acids; SFA: saturated fatty acids. BWHHS: British Women's Heart and Health Study; BRHS: British Regional Heart Study; WHII: Whitehall-II Study; SABRE: Southall And Brent REvisited cohort; UKCTOCS: United Kingdom Collaborative Trial of Ovarian Cancer Screening; CaPS: Caerphilly Prospective Study; CHD: coronary heart disease.

Table S3. Association of blood fatty acids concentration with demographic and lifestyle factors

	N (range)	DHA					LA					MUFA					SFA				
		Beta	95% CI		p	I^2	Beta	95% CI		p	I^2	Beta	95% CI		p	I^2	Beta	95% CI		p	I^2
Non-European	19,928-20,025	-0.02	-0.46	0.42	0.925	95	0.15	0.08	0.21	6E-06	0	-0.42	-0.67	-0.17	0.001	83	-0.25	-0.44	-0.06	0.009	70
Age (> 65 years)	20,047-20,144	0.11	0.03	0.18	0.006	78	-0.01	-0.08	0.05	0.731	69	-0.02	-0.07	0.04	0.522	55	-0.02	-0.08	0.05	0.607	66
Ever smokers	14,828-14,888	-0.05	-0.28	0.17	0.642	96	-0.11	-0.16	-0.07	1E-07	0	0.30	0.22	0.37	3E-13	70	0.12	0.04	0.21	0.006	76
Alcohol drinkers	14,906-14,965	0.35	0.2	0.49	3E-06	92	-0.08	-0.12	-0.04	7E-05	0	0.18	0.00	0.35	0.053	95	0.11	0.05	0.17	0.001	56
Overweight/obese	19,402-19,498	0.04	-0.03	0.11	0.270	83	0.02	-0.04	0.08	0.475	72	0.37	0.29	0.45	0.000	87	0.26	0.15	0.38	9E-06	94

Beta: study- and sex-specific standard deviation units of blood fatty acids concentration according to study covariates. DHA: docosahexaenoic acid; LA: linoleic acid; MUFA: monounsaturated fatty acids; SFA: saturated fatty acids.

Table S4. Correlation across circulating fatty acids

	DHA						LA						MUFA					
	BWHHS	BRHS	WHII	SABRE	CaPS	UKCTOCS	BWHHS	BRHS	WHII	SABRE	CaPS	UKCTOCS	BWHHS	BRHS	WHII	SABRE	CaPS	UKCTOCS
DHA	1	1	1	1	1	1												
LA	0.49	0.36	0.28	0.36	0.40	0.32	1	1	1	1	1	1						
MUFA	0.36	0.36	0.40	0.42	0.45	0.30	0.42	0.45	0.51	0.38	0.43	0.51	1	1	1	1	1	1
SFA	0.52	0.45	0.42	0.25	0.53	0.37	0.67	0.70	0.71	0.16	0.71	0.68	0.82	0.86	0.88	0.50	0.86	0.88

Pearson’s correlation coefficient (all P-values < 0.0001).

Table S5. Pooled estimates and heterogeneity metrics for meta-analysis of the association of circulating fatty acids with risk of coronary heart disease

Fatty acid	Model	OR	OR - 95% CI		P	Cochrane's Q	df	P for Cochrane's Q	I^2	I^2 - 95% reference interval*	
DHA	M0	0.92	0.82	1.04	0.206	30	5	1.21E-05	84	66	92
DHA	M1	0.94	0.82	1.07	0.340	32	5	4.80E-06	85	68	93
DHA	M2	0.85	0.76	0.95	0.005	17	5	0.005	70	29	87
LA	M0	1.05	0.92	1.20	0.480	40	5	1.48E-07	88	75	94
LA	M1	1.05	0.92	1.20	0.451	40	5	1.87E-07	87	75	94
LA	M2	1.01	0.87	1.18	0.854	24	5	2.36E-04	79	54	90
MUFA	M0	1.20	1.12	1.28	2E-07	11	5	0.047	55	0	82
MUFA	M1	1.18	1.13	1.24	4E-13	4	5	0.609	0	0	75
MUFA	M2	1.36	1.15	1.61	4E-04	17	5	0.005	70	31	87
SFA	M0	1.12	1.05	1.20	0.001	12	5	0.035	58	0	83
SFA	M1	1.12	1.05	1.20	0.001	10	5	0.065	52	0	81
SFA	M2	0.94	0.82	1.09	0.421	9	5	0.100	46	0	79

Model 0 (M0): unadjusted model; Model 1 (M1): adjusted for recruitment place, demographic and lifestyle variables (age, sex, non-European ancestry, smoking, alcohol and body mass index); Model 2 (M2): adjusted for variables in M1 plus other fatty acids. DHA: docosahexaenoic acid; LA: linoleic acid; MUFA: monounsaturated fatty acids; SFA: saturated fatty acids. * I^2 and its 95% reference interval were estimated as proposed by Higgins et al [8].

Table S6. Pooled estimates and heterogeneity metrics for meta-analysis of the association of circulating fatty acids with risk of stroke

Fatty acid	Model	OR	OR - 95% CI		P	Cochrane's Q	df	P for Cochrane's Q	I^2	I^2 - 95% reference interval*	
DHA	M0	0.93	0.87	0.99	0.022	4.0	5	0.554	0	0	75
DHA	M1	0.93	0.87	0.99	0.016	4.8	5	0.447	0	0	75
DHA	M2	0.95	0.89	1.02	0.165	3.4	5	0.641	0	0	75
LA	M0	0.85	0.78	0.92	1E-04	8.4	5	0.136	40	0	76
LA	M1	0.85	0.78	0.93	2E-04	8.7	5	0.124	42	0	77
LA	M2	0.82	0.75	0.90	3E-05	5.7	5	0.339	12	0	78
MUFA	M0	1.03	0.96	1.12	0.405	7.6	5	0.182	34	0	73
MUFA	M1	1.04	0.98	1.11	0.186	5.2	5	0.390	4	0	76
MUFA	M2	1.22	1.03	1.44	0.022	9.4	5	0.092	47	0	79
SFA	M0	0.96	0.88	1.04	0.308	9.1	5	0.104	45	0	78
SFA	M1	0.96	0.89	1.04	0.353	7.0	5	0.220	29	0	71
SFA	M2	0.94	0.79	1.11	0.451	7.6	5	0.178	34	0	74

Model 0 (M0): unadjusted model; Model 1 (M1): adjusted for recruitment place, demographic and lifestyle variables (age, sex, non-European ancestry, smoking, alcohol and body mass index); Model 2 (M2): adjusted for variables in M1 plus other fatty acids. DHA: docosahexaenoic acid; LA: linoleic acid; MUFA: monounsaturated fatty acids; SFA: saturated fatty acids. * I^2 and its 95% reference interval were estimated as proposed by Higgins et al [8].

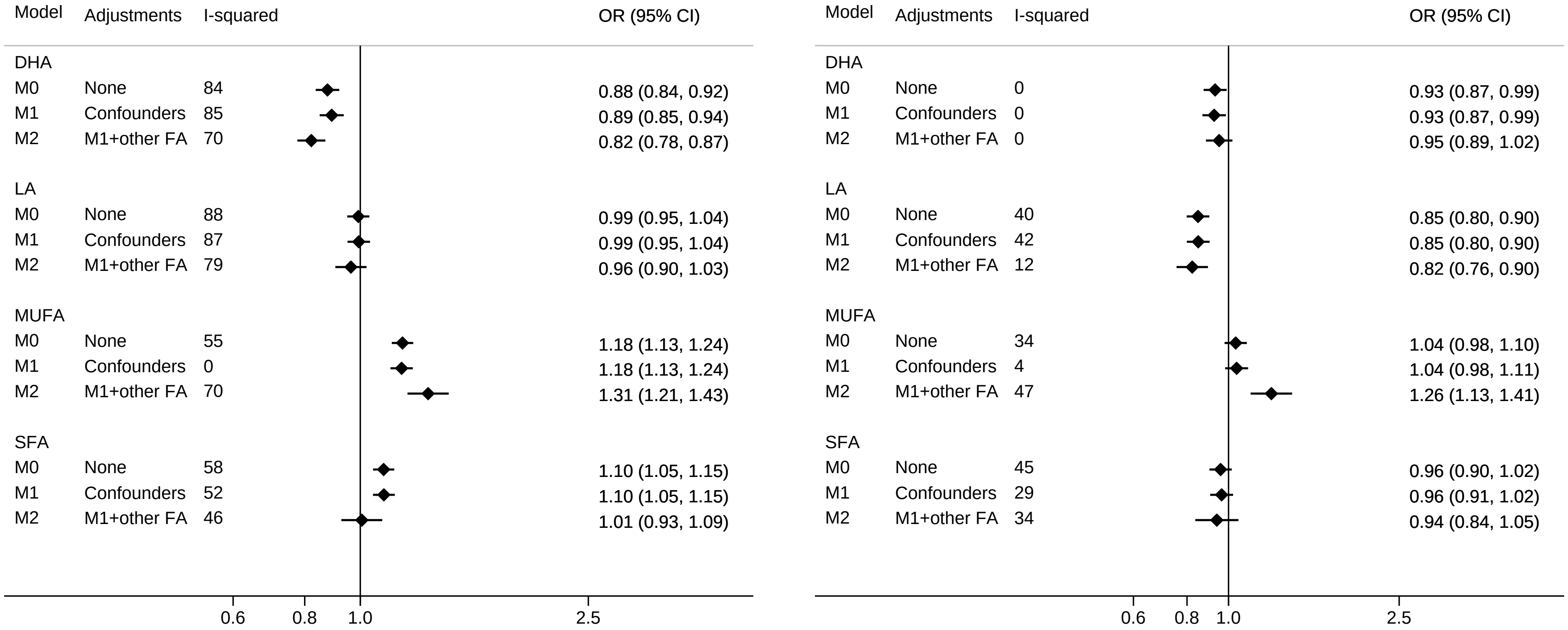

Figure S1. Odds ratio for coronary heart disease (CHD) and stroke according to blood fatty acids concentration (fixed effects meta-analysis)

Results were pooled using fixed effect meta-analysis and are expressed as odds ratio (and 95% confidence interval) per standard deviation unit increase in blood fatty acids concentration. Each standard unit corresponds to approximately 0.06 mmol/L for DHA, 0.7 for LA, 1.0 for MUFA, and 1.1 for SFA. Model 0 (M0): unadjusted model; Model 1 (M1): adjusted for recruitment place, demographic and lifestyle variables (age, sex, non-European ancestry, smoking, alcohol and body mass index); Model 2 (M2): adjusted for variables in M1 plus other fatty acids. DHA: docosahexaenoic acid; LA: linoleic acid; MUFA: monounsaturated fatty acids; SFA: saturated fatty acids; CHD: coronary heart disease.

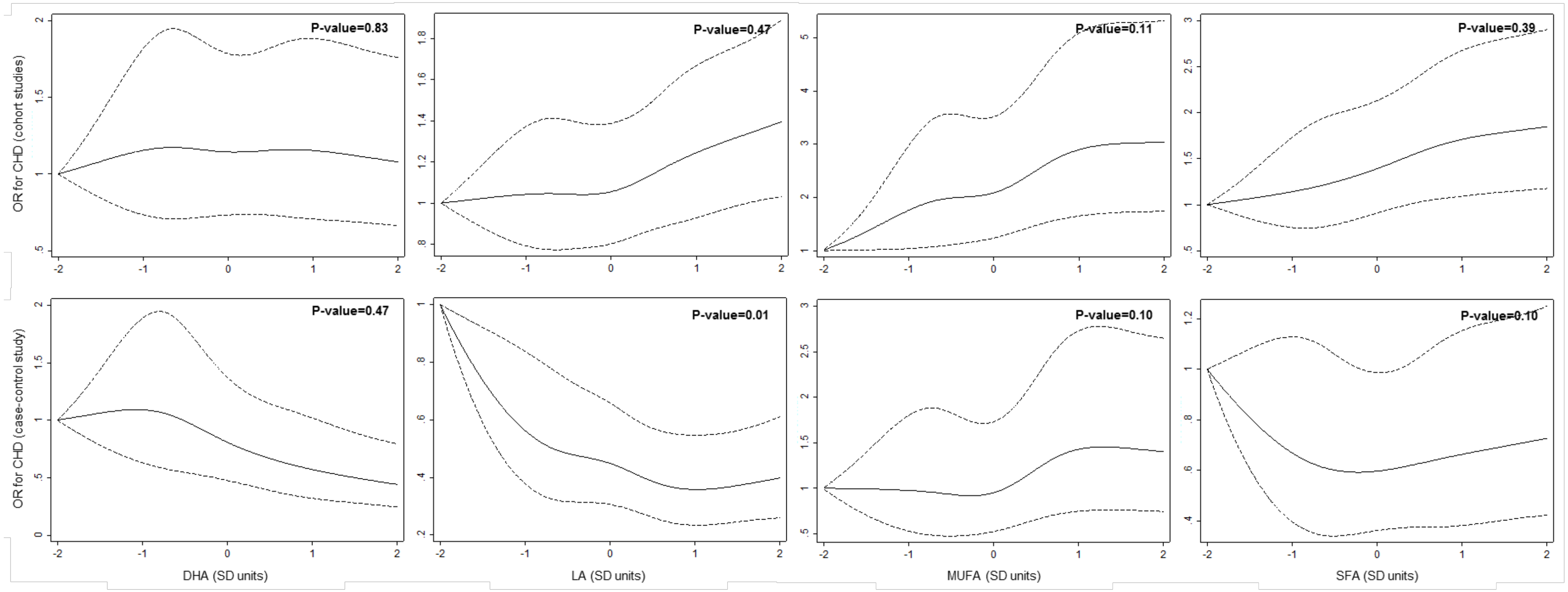

Figure S2. Dose-response curve for the association between blood fatty acids and coronary heart disease (CHD) risk

Results are expressed as odds ratio (and 95% confidence interval) according blood fatty acids concentration in standard deviation units. Each standard unit corresponds to approximately 0.06 mmol/L for DHA, 0.7 for LA, 1.0 for MUFA, and 1.1 for SFA. Models were adjusted for recruitment place, demographic and lifestyle variables (age, sex, non-European ancestry, smoking, alcohol and body mass index). P-value for the comparison between linear and nonlinear (restricted cubic spline) models were derived from the likelihood-ratio test. P-value threshold after Bonferroni correction = 0.05/16 = 0.003. DHA: docosahexaenoic acid; LA: linoleic acid; MUFA: monounsaturated fatty acids; SFA: saturated fatty acids. The relations of the fatty acids with CHD risk were also modelled using blood fatty acids concentration in mmol/L (not in study-specific standard deviation units) and conclusions were the similar (cohort studies: P_{DHA}= 0.41, P_{LA}= 0.32, P_{MUFA}= 0.29, P_{SFA}= 0.10; case-control study: P_{DHA}= 0.46, P_{LA}= 0.01, P_{MUFA}= 0.05, P_{SFA}= 0.09).

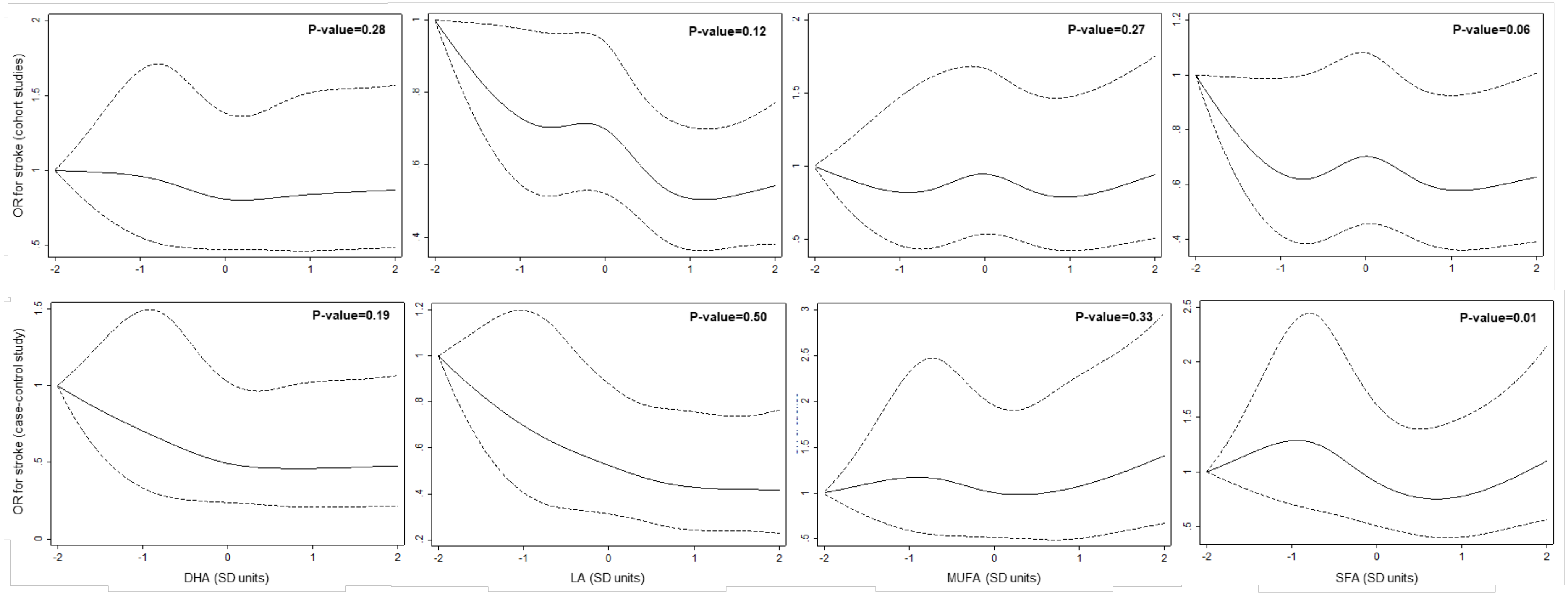

Figure S3. Dose-response curve for the association between blood fatty acids and stroke risk

Results are expressed as odds ratio (and 95% confidence interval) according blood fatty acids concentration in standard deviation units. Each standard unit corresponds to approximately 0.06 mmol/L for DHA, 0.7 for LA, 1.0 for MUFA, and 1.1 for SFA. Models were adjusted for recruitment place, demographic and lifestyle variables (age, sex, non-European ancestry, smoking, alcohol and body mass index). P-value for the comparison between linear and nonlinear (restricted cubic spline) models were derived from the likelihood-ratio test. P-value threshold after Bonferroni correction = 0.05/16 = 0.003. DHA: docosahexaenoic acid; LA: linoleic acid; MUFA: monounsaturated fatty acids; SFA: saturated fatty acids. The relations of the fatty acids with stroke risk were also modelled using blood fatty acids concentration in mmol/L (not in study-specific standard deviation units) and conclusions were similar (cohort studies: P_{DHA}= 0.69, P_{LA}= 0.12, P_{MUFA}= 0.80, P_{SFA}= 0.30; case-control study: P_{DHA}= 0.22, P_{LA}= 0.52, P_{MUFA}= 0.30, P_{SFA}= 0.01).

Table S7. P-value for the association between the complete case indicator for CHD or stroke analyses and study variables

	BWHHS		BRHS		WH2		SABRE		CaPS		UKCTOCS	
	P-value (CHD)	P-value (stroke)	P-value (CHD)	P-value (stroke)	P-value (CHD)	P-value (stroke)	P-value (CHD)	P-value (stroke)	P-value (CHD)	P-value (stroke)	P-value (CHD)	P-value (stroke)
Age (y)	8.83E-05	8.83E-05	0.33	0.33	0.61	0.09	0.01	3.57E-03	2.17E-04	2.17E-04	0.90	0.07
Sex	—	—	—	—	0.47	0.49	5.04E-76	1.11E-74	—	—	—	—
European ancestry	0.93	0.93	0.41	0.41	—	—	6.48E-36	1.30E-34	—	—	0.17	—
Smoking	1.55E-05	1.55E-05	0.82	0.82	2.67E-08	1.79E-05	0.69	0.63	0.95	0.95	—	—
Alcohol drinking	0.38	0.38	0.48	0.48	0.47	0.08	2.96E-21	6.20E-20	0.27	0.27	—	—
BMI	1.52E-05	1.52E-05	3.60E-04	3.60E-04	0.01	1.94E-03	4.74E-06	4.29E-06	—	—	0.53	0.90
DHA	3.47E-04	3.47E-04	8.18E-04	8.18E-04	0.53	0.67	0.15	0.32	9.90E-05	9.90E-05	8.39E-07	3.82E-05
LA	3.06E-03	3.06E-03	0.25	0.25	0.63	0.55	0.16	0.11	0.96	0.96	3.20E-04	1.09E-07
MUFA	1.17E-07	1.17E-07	1.04E-03	1.04E-03	0.05	0.05	0.95	0.78	0.35	0.35	2.01E-03	2.62E-05
SFA	1.54E-06	1.54E-06	2.99E-03	2.99E-03	0.27	0.27	0.30	0.61	0.49	0.49	4.56E-05	1.02E-07
CHD	0.03	—	0.89	—	0.16	—	1.69E-15	—	0.11	—	1.04E-04	—
Stroke	—	3.13E-03	—	0.81	—	0.59	—	0.04	—	0.57	—	2.37E-03

Table S8. Odds ratio for coronary heart disease (CHD) and stroke according to blood fatty acids concentration after multiple imputation

	CHD		Stroke	
	**OR**	**95% CI**	**OR**	**95% CI**
**DHA**				
M0	0.92	(0.82; 1.03)	0.94	(0.89; 1.00)
M1	0.95	(0.83; 1.08)	0.94	(0.88; 0.99)
M2	0.87	(0.77; 0.97)	0.96	(0.90; 1.03)
**LA**				
M0	1.03	(0.90; 1.17)	0.86	(0.80; 0.92)
M1	1.03	(0.90; 1.17)	0.86	(0.80; 0.93)
M2	0.98	(0.86; 1.12)	0.82	(0.76; 0.89)
**MUFA**				
M0	1.19	(1.11; 1.27)	1.04	(0.98; 1.11)
M1	1.17	(1.12; 1.22)	1.04	(0.98; 1.11)
M2	1.35	(1.14; 1.61)	1.18	(1.00; 1.40)
**SFA**				
M0	1.12	(1.04; 1.20)	0.97	(0.89; 1.05)
M1	1.11	(1.03; 1.20)	0.97	(0.90; 1.05)
M2	0.93	(0.80; 1.09)	0.96	(0.82; 1.12)

Results are expressed as odds ratio (and 95% confidence interval) per standard deviation unit increase in blood fatty acids concentration. Model 0 (M0): unadjusted model; Model 1 (M1): adjusted for recruitment place, demographic and lifestyle variables (age, sex, non-European ancestry, smoking, alcohol and body mass index); Model 2 (M2): adjusted for variables in M1 plus other fatty acids. DHA: docosahexaenoic acid; LA: linoleic acid; MUFA: monounsaturated fatty acids; SFA: saturated fatty acids; CHD: coronary heart disease.

References

1. Soininen P, Kangas AJ, Wurtz P, Tukiainen T, Tynkkynen T, Laatikainen R, Jarvelin MR, Kahonen M, Lehtimaki T, Viikari J, Raitakari OT, Savolainen MJ and Ala-Korpela M. High-throughput serum NMR metabonomics for cost-effective holistic studies on systemic metabolism. *Analyst*. 2009;134:1781-5.
2. Inouye M, Kettunen J, Soininen P, Silander K, Ripatti S, Kumpula LS, Hamalainen E, Jousilahti P, Kangas AJ, Mannisto S, Savolainen MJ, Jula A, Leiviska J, Palotie A, Salomaa V, Perola M, Ala-Korpela M and Peltonen L. Metabonomic, transcriptomic, and genomic variation of a population cohort. *Mol Syst Biol*. 2010;6:441.
3. Soininen P, Kangas AJ, Wurtz P, Suna T and Ala-Korpela M. Quantitative serum nuclear magnetic resonance metabolomics in cardiovascular epidemiology and genetics. *Circ Cardiovasc Genet*. 2015;8:192-206.
4. Kujala UM, Makinen VP, Heinonen I, Soininen P, Kangas AJ, Leskinen TH, Rahkila P, Wurtz P, Kovanen V, Cheng S, Sipila S, Hirvensalo M, Telama R, Tammelin T, Savolainen MJ, Pouta A, O'Reilly PF, Mantyselka P, Viikari J, Kahonen M, Lehtimaki T, Elliott P, Vanhala MJ, Raitakari OT, Jarvelin MR, Kaprio J, Kainulainen H and Ala-Korpela M. Long-term leisure-time physical activity and serum metabolome. *Circulation*. 2013;127:340-8.
5. Vehtari A, Makinen VP, Soininen P, Ingman P, Makela SM, Savolainen MJ, Hannuksela ML, Kaski K and Ala-Korpela M. A novel Bayesian approach to quantify clinical variables and to determine their spectroscopic counterparts in 1H NMR metabonomic data. *BMC Bioinformatics*. 2007;8 Suppl 2:S8.
6. Würtz P, Kangas A, Soininen P, Lawlor D, Davey Smith G and Ala-Korpela M. Quantitative serum NMR metabolomics in large-scale epidemiology: a primer on -omic technology. *American Journal of Epidemiology*. 2017.
7. Wurtz P, Havulinna AS, Soininen P, Tynkkynen T, Prieto-Merino D, Tillin T, Ghorbani A, Artati A, Wang Q, Tiainen M, Kangas AJ, Kettunen J, Kaikkonen J, Mikkila V, Jula A, Kahonen M, Lehtimaki T, Lawlor DA, Gaunt TR, Hughes AD, Sattar N, Illig T, Adamski J, Wang TJ, Perola M, Ripatti S, Vasan RS, Raitakari OT, Gerszten RE, Casas JP, Chaturvedi N, Ala-Korpela M and Salomaa V. Metabolite profiling and cardiovascular event risk: a prospective study of 3 population-based cohorts. *Circulation*. 2015;131:774-85.
8. Higgins JP and Thompson SG. Quantifying heterogeneity in a meta-analysis. *Stat Med*. 2002;21:1539-58.
